# Effect of Chinese eye exercises on change in visual acuity and eyeglasses wear among school-aged children in rural China: a propensity-score-matched cohort study

**DOI:** 10.1186/s12906-020-2878-9

**Published:** 2020-03-13

**Authors:** Huan Wang, Yiwei Qian, Nathan Congdon, Matthew Boswell, Scott Rozelle, Xiaochen Ma

**Affiliations:** 1 Freeman Spogli Institute of International Studies, Stanford University, Stanford, CA USA; 2 Department of Economics, University of Southern California, Los Angeles, CA USA; 3 Centre for Public Health, Queen’s University Belfast, Belfast, UK; 4 Zhongshan Ophthalmic Center, Sun Yat-sen University, Guangzhou, China; 5 China Center for Health Development Studies, Peking University, No 38 Xuyuan Road, Haidian District, Beijing, 100191 China

**Keywords:** Eye exercises, Visual acuity, Eyeglasses wear, Chinese children

## Abstract

**Background:**

Daily “eye exercises,” massaging of periocular acupuncture pressure points, have been part of China’s national vision care policy in schools for some 50 years. However, the effect of eye exercises on myopia progression and eyeglasses wear has not been definitively investigated. This study evaluates the effectiveness of eye exercises on visual acuity and the propensity of rural children to wear eyeglasses.

**Methods:**

Cohort study in 252 randomly-selected rural schools with baseline in September 2012 and follow up surveys 9 and 21 months later. Outcomes were assessed using propensity-score matching (PSM), multivariate linear regression and logistic regression to adjust for differences between children performing and not performing eye exercises.

**Results:**

Among 19,934 children randomly selected for screening, 2374 myopic (spherical equivalent refractive error ≤ − 0.5 diopters in either eye) children (11.9%, mean age 10.5 [Standard Error 1.08] years, 48.5% boys) had VA in either eye ≤6/12 without eyeglasses correctable to > 6/12 with eyeglasses. Among these who completed the 21-month follow up, 1217 (58.2%) children reported practicing eye exercises on school days and 874 (41.8%) did not. After propensity-score matching, 1652 (79%) children were matched: 826 (50%) in the Eye Exercises group and 826 (50%) in the No Exercise group. Performing eye exercises was not associated with change in LogMAR uncorrected visual acuity and wear of eyeglasses, using either logistic regression or PSM at 9 or 21 months.

**Conclusions:**

We found no evidence for an effect of eye exercises on change in vision or eyeglasses wear.

**Trial registration:**

The original trial (Registration site: http://isrctn.org. Registration number: ISRCTN03252665) was retrospectively registered 25/09/2012.

## Background

Poor vision accounts for nearly half of all disability among children in the developing world [1]. The most common and easily-treated cause of visual impairment (visual acuity < 6/18) is refractive error, which affects 12.8 million children between the ages of 5 and 15 globally [2]. Approximately one half of these children reside in China [3, 4]. Spectacles provide safe and inexpensive correction for refractive error, but among children requiring correction in rural and urban migrant populations in China, spectacle wear is as low as 15% [5–7]. The situation is due in part to a wide-spread perception among parents, teachers and even some healthcare providers that eyeglasses wear harms children’s vision by worsening myopia [8, 9].

In 1963, the Ministry of Education introduced Chinese eye exercises as a proposed method for myopia treatment and/or prevention among school-aged children [10]. In Chinese eye-exercises, pressure is applied to acupoints present around the eyes, with the objective of increasing blood circulation, reducing ocular fatigue, and slowing down the development of myopia [11].

Eye exercises are meant to be done in all schools throughout the country according to a nationally-designated protocol. Students are supposed to carry out the exercises at least once each school day (that is, 5 times per week). Each eye exercise session is 5 min long, and these are generally not overseen by school instructors. The same exercises are recommended for all students, regardless of the presence or absence of refractive error.

However, the effect of eye exercises on myopia incidence and progression is not well understood, as shown in a recent Cochrane review [12]. There are few studies of the association between eye exercises and students’ vision, mostly employing small sample sizes with short term follow up. These have generally found no clinically significant association between correctly-performed eye exercises and a reduction in accommodative lag in children, a factor implicated in myopia progression [10]. One study reported no association between eye exercises and the risk of myopia onset or myopia progression [11]. A significant concern for confounding in existing studies lies in the fact that children choosing to practice eye exercises and families encouraging such behavior may differ in important ways from those who do not, including possible differences in refractive error and its determinants. A randomized controlled trial (RCT) design would be ideal to assess the possible causal association between eye exercises and myopia, but given the widespread belief in the efficacy of this intervention, randomizing children to a non-exercise control group would likely not be considered ethical in China. The best evidence is likely to come from well-designed observational studies, which adequately control for potential confounding variables and monitor refractive error in addition to visual acuity as outcomes, to investigate the effect of Chinese eye exercises as they are performed in schools. Though schools are generally meant to require students to carry out eye exercises, evidence of wide variation in compliance at the individual level [13, 14] leaves open the possibility of studying their effectiveness using other statistical techniques besides random assignment, which can help to reduce or account for differences between children who do and do not take part.

In this paper, we aim to investigate the association between self-reported regular practice of eye exercises and change in uncorrected visual acuity (a surrogate for myopia) over the course of two school years, using propensity score matching to account for differences between compliant and non-compliant children. This represents a post hoc analysis of data from a large, cluster-randomized, population-based trial on the educational impact of providing eyeglasses to school-aged children with refractive error, carried out in two provinces in rural China [7]. Our secondary outcome, wear of free spectacles distributed during the parent trial, examines the hypothesis that children practicing eye exercises may be less likely to use eyeglasses if they own them.

## Methods

The protocol for this study was approved in full by Institutional Review Boards at Stanford University (Palo Alto, California, USA) and the Zhongshan Ophthalmic Center of Sun Yat-sen University (ZOC, Guangzhou, China). Permission was received from local Boards of Education in each region and from the principals of all schools. The principles of the Declaration of Helsinki were followed throughout. Written informed consent was obtained from at least one parent for all child participants. The original trial was designed to study the effect of providing free spectacles on children’s educational performance, and found that scores on a study-specific mathematic test were statistically significantly higher in the group randomized to receive free spectacles compared to controls [7].

Practicing eye exercises regularly, if it does in fact reduce progression of myopia, would be expected to slow the decline in uncorrected distance visual acuity (VA) resulting from increased myopia commonly observed among children with aging. The primary outcome of the current study is the change in between-group difference in uncorrected VA over the course of one (9 months) and two (21 months) school years (using a difference in difference methodology). The choice of this outcome is based on the fact that vision itself, rather than refractive power, is the outcome of interest from the standpoint of disability and its alleviation [9]. The methods of the original trial have been described previously [7] and are summarized here for reference.

### Setting, sampling, and eligibility criteria

The study was carried out in two locations in western China: one prefecture in Gansu, one of China’s poorest provinces; and a neighboring and more affluent prefecture in Shaanxi, a middle-income province [15]. One school from each of the townships in both prefectures was randomly selected from a list of all primary schools, and within each school, one class was randomly chosen in each of the fourth and fifth grades (expected age 12 to 14 years). All children at the 252 selected schools were eligible if they had an uncorrected (without eyeglasses) visual acuity of ≤6/12 in either eye, correctable with eyeglasses to > 6/12 in both eyes, and their refractive error was ≤ − 0.50 diopters (that is, myopic). The relatively small number of students with hyperopia and astigmatism were excluded from this study.

### Questionnaires and assessment of compliance with eye exercises

At baseline (September 2012, the beginning of the school year), enumerators administered questionnaires developed for this study to the sample children, collecting information on the child’s age, sex, wear of eyeglasses, time spent out of doors and time engaged in near/middle distance work (potential determinants of myopia progression) [16], and parental migration, education and eyeglasses wear (see additional supplementary files 1 and 2 for questionnaire used in the survey). A parental questionnaire asked about ownership of 13 selected items as an index of family wealth. During follow up surveys (June 2013, 9 months later and May 2014, 21 months later) children again filled out a questionnaire on eyeglasses wear.

At the time of the baseline and 9-month follow up surveys, all children answered the question “do you practice eye exercises regularly,” as either “yes” or “no.” Children who reported practicing eye exercises regularly at baseline were categorized in the “eye exercises” group in the 9 months analysis, and children performing exercises at both baseline and at 9 months were categorized in the “eye exercises” group in the 21 months analysis.

### Assessment of visual acuity and refraction

Children underwent baseline VA screening at school by an eye nurse and staff assistant, previously trained by optometrists from ZOC. VA was tested separately for each eye without refractive correction at 4 m using Early Treatment Diabetic Retinopathy Study (ETDRS) charts [17] (Precision Vision, La Salle, Illinois, USA) in a well-lighted, indoor area. If at least 4 of 5 optotypes on the LogMAR 1.0 (Snellen 6/60) line were correctly identified, children were examined on the LogMAR 0.70 (Snellen 6/30) line, the LogMAR 0.40 (Snellen 6/15) line, and then line by line to LogMAR − 0.30 (Snellen 6/3). If a line was failed, lines above were tested successively until the child identified 4 of 5 optotypes, with the VA for an eye defined as the lowest line read successfully. If the top line could not be read at 4 m, the subject was tested as above at 1 m, and the measured Snellen VA value was divided by 4, the equivalent of adding 0.6 to the measured LogMAR acuity.

Children with uncorrected VA ≥ LogMAR 0.3 (≤ Snellen 6/12) in either eye underwent cycloplegia with two drops of 1% cyclopentolate in each eye, delivered at 5 min intervals, after anesthesia with 0.5% proparacaine. After an additional 30 min, if the pupillary light reflex was still present, a third round of both cyclopentolate and proparacaine was administered. After a further 15–20 min interval, students whose pupils were considered fully dilated (≥ 6 mm in diameter with absent pupillary light reflex) underwent automated refraction (Topcon KR 8900; Tokyo, Japan), with subjective refinement by a refractionist, previously trained by experienced pediatric optometrists from ZOC.

### Outcome assessment

The primary outcome of the current analysis is the difference in change in uncorrected VA between the groups who did and did not report regularly practicing eye exercises over the course of one (9 months) to two (21 months) school years. During the follow up surveys, at 9 and 21 months after the baseline, VA was re-assessed using the same protocol and vision chart as described above. Eyeglass wear was assessed through unannounced direct examinations carried out by study personnel at the schools at 9 and 21 months.

### Statistical methods

Family wealth was calculated by summing the value, as reported in the China Rural Household Survey Yearbook [18], of items on a list of 13 that were owned by the family. Refractive power was defined throughout as the spherical equivalent, the spherical power plus half the cylindrical power.

Change in uncorrected VA was calculated by subtracting the uncorrected VA (logarithm of the minimal angle of resolution [LogMAR]) at 9 and 21 months follow up from the baseline uncorrected VA. Change in uncorrected VA over time was then compared between children that either did and did not regularly perform eye exercises.

Our primary analysis also used propensity score matching (PSM) [14, 19]. PSM is a strategy that attempts to correct for selection bias, providing an alternative for estimating treatment effects when systematic differences between groups are not random [14, 20]. The propensity score is a measure of the likelihood that a student would perform eye exercises regularly using the student’s covariate scores. Matching techniques work by matching the baseline characteristics of children not performing eye exercises regularly with those who did. Potential determinants of myopia used to create propensity scores included children’s age, sex, spherical equivalent refractive power in the more myopic eye, VA at baseline, ownership of eyeglasses, time spent on near work, middle distance activities and outdoor activity, and parental ownership of eyeglasses, migration and education [7, 16, 21]. Children with the same propensity scores were matched one to one without replacement, making the two groups equal in size by excluding non-matching cases.

Multivariate linear regression and PSM were used to estimate the impact of eye exercises on change in uncorrected VA (as an index of myopia progression). Logistic regression and PSM were used to estimate the impact of eye exercises on eyeglasses wear. The simplest regression model included only change in uncorrected VA from baseline to 9 and 21 months in the better-seeing eye (outcome) and self-reported regular participation in eye exercises (independent variable). Other baseline variables were also investigated as predictors of final VA, with the final model including intervention arm in the original trial and variables associated with baseline VA at *P* ≤ 0.20, potentially including children’s baseline age, sex, refractive power in the most myopic eye, VA, ownership of eyeglasses, time spent on near work, middle distance activities and outdoor activity, parental ownership of eyeglasses, migration and education, and family wealth.

We calculated the standardized difference between the eye exercise groups [22], that is the mean difference divided by the pooled standard deviation, expressed as a percentage, and reported 95% confidence intervals (CI) around this value. Standardized differences were estimated for all covariates before and after matching to assess pre- and post-match balance.

All analyses were performed using Stata 14.0 (Stata Corp, College Station, Texas, USA.)

## Results

Among 19,934 children randomly selected for screening, 2374 meeting myopia and visual criteria (11.9%, mean age 10.5 years, 1152 boys [48.5%]) were eligible. Among them, 2091 children completed the 21 month follow up, while 1217 (58.2%) children practiced eye exercises regularly and 874 (41.8%) did not. Their baseline characteristics are described in Table 1.

**Table 1:** Baseline characteristics of children with and without the regular practice of eye exercises, full sample

Characteristic	Regularly Practiced Eye Exercises	Did Not Regularly Practice Eye Exercises	Standardized Difference^a^, %	*P* Value^b^	Differences Between Means 95% CI
	*n* = 1217	*n* = 874			
Age, years, mean (SD)	10.40 (1.05)	10.48 (1.18)	−7.6	0.240	(−0.228, 0.058)
Data missing	2 (0.2)	0			
Male, n (%)	596 (48.56)	428 (48.62)	−0.1	0.977	(− 0.044, 0.043)
Baseline myopia, diopters, mean (SD)	−3.14 (0.95)	−3.18 (0.95)	− 3.8	0.435	(−0.128, 0.055)
Baseline uncorrected VA, mean (SD)	0.53 (0.21)	0.54 (0.23)	−2.0	0.704	(−0.027, 0.018)
Have eyeglasses at baseline, n (%)^c^	170 (14.05)	122 (13.84)	0.6	0.901	(−0.031, 0.035)
Received free eyeglasses, n(%)^d^	815 (66.88)	577 (66.13)	1.6	0.894	(−0.104, 0.119)
Near work, h/wk, mean (SD)	7.21 (3.58)	7.42 (3.74)	−5.6	0.298	(−0.593, 0.183)
Data missing	1 (0.1)	0			
Middle distance work, h/wk, mean (SD)	4.86 (4.11)	4.95 (4.25)	−2.2	0.688	(−0.535, 0.354)
Data missing	2 (0.2)	2 (0.2)			
Outdoor time, h/wk, mean (SD)	8.18 (3.94)	7.94 (3.98)	6.0	0.329	(−0.243, 0.722)
Data missing	3 (0.2)	1 (0.1)			
≥ 1 parent wears eyeglasses, n (%)	401 (33.03)	340 (38.87)	−12.2	0.010	(−0.103, −0.014)
Data missing	3 (0.2)	2 (0.2)			
≥ 1 Parent with > 12 y of education, n (%)	241 (19.53)	181 (21.29)	−4.4	0.392	(−0.058, 0.023)
Data missing	14 (1.2)	10 (1.1)			
Both parents out-migrated for work, n(%)	133 (10.62)	95 (11.45)	−2.7	0.528	(−0.034, 0.018)
Data missing	12 (1.0)	10 (1.1)			
Family wealth index, mean (SD)	2.16 (0.76)	1.97 (0.75)	25.0	0.000	(0.104, 0.275)
Data missing	43 (3.5)	30 (3.4)			

*Abbreviation*: *VA* visual acuity. Measured in LogMAR (logarithm of the Minimum Angle of Resolution). Change in LogMAR of 0.1 indicates 1 line change on the vision chart

^a^Standardized difference is the mean difference divided by the pooled standard deviation, expressed as a percentage

^b^Cluster effects within school were adjusted for in all comparisons

^c^Defined as having eyeglasses at school at baseline, having previously been told to bring them

^d^Defined as being enrolled in a treatment school that received free eyeglasses

**Fig. 1:**
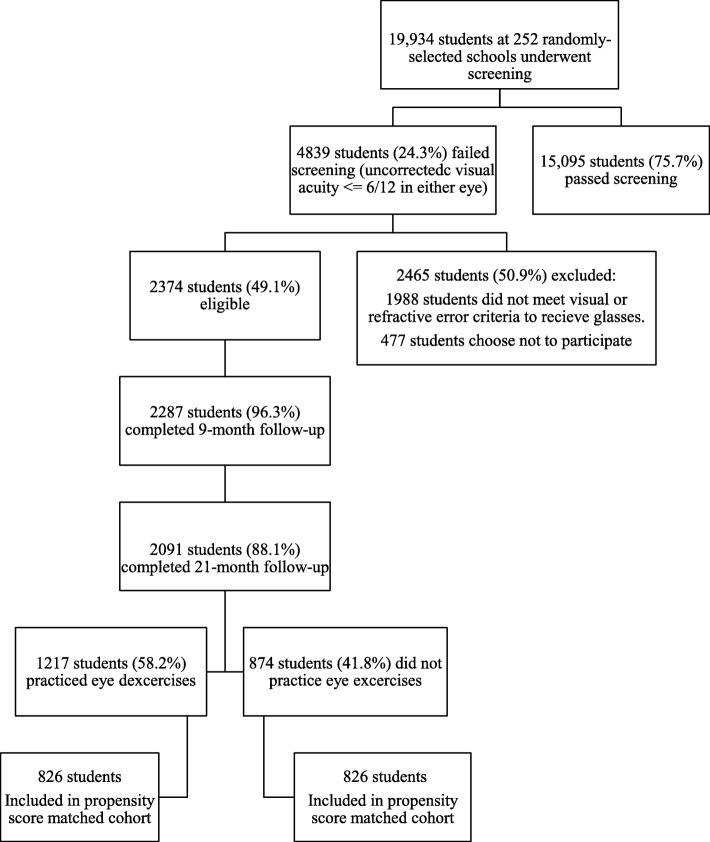
Flow Chart of Subjects in Study

Propensity-score matching (PSM) was employed among these 2091 children, with 1652 (79%) children matched successfully, 826 (50%) in the eye exercises group and 826 (50%) in the no eye exercises group (Fig. 1). Table 2 shows that after matching, there were no significant differences between the two groups in baseline characteristics.

**Table 2:** Baseline characteristics of children with and without self-reported regular practice of eye exercises, matched sample

Characteristic	Regularly Practiced Eye Exercises	Did Not Regularly Practice Eye Exercises	Standardized Difference^a^, %	*P* Value^b^	Differences Between Means 95% CI
	*n* = 826	n = 826			
Age, years, mean (SD)	10.38 (1.08)	10.49 (1.18)	−9.1	0.206	(−0.260, 0.056)
Male, n (%)	404 (48.91)	402 (48.67)	0.5	0.920	(−0.045, 0.050)
Baseline myopia, diopters, mean (SD)	−3.21 (0.91)	−3.18 (0.95)	2.9	0.564	(−0.067, 0.123)
Baseline uncorrected VA, mean (SD)	0.54 (0.20)	0.53 (0.22)	4.4	0.438	(−0.015, 0.034)
Have eyeglasses at baseline, n (%)^c^	114 (13.80)	113 (13.68)	0.3	0.945	(−0.034, 0.036)
Received free eyeglasses, n (%)^d^	539 (64.04)	541 (65.49)	−3.1	0.802	(−0.128, 0.099)
Near work, h/wk, mean (SD)	7.78 (3.63)	7.41 (3.75)	10.4	0.072	(− 0.034, 0.785)
Middle distance work, h/wk, mean (SD)	5.34 (4.35)	4.98 (4.30)	8.5	0.149	(− 0.128, 0.836)
Outdoor time, h/wk, mean (SD)	8.11 (4.01)	7.91 (3.94)	5.2	0.409	(−0.281, 0.689)
≥ 1 parent wears eyeglasses, n (%)	365 (44.19)	322 (38.98)	10.8	0.054	(−0.001, 0.105)
≥ 1 Parent with > 12 y of education, n (%)	174 (21.06)	176 (21.30)	−0.6	0.912	(−0.046, 0.041)
Both parents out-migrated for work n (%)	83 (10.05)	95 (11.50)	−4.6	0.344	(−0.045, 0.016)
Family wealth index, mean (SD)	2.00 (0.77)	1.97 (0.75)	3.4	0.554	(−0.059, 0.110)

*Abbreviation*: *VA* visual acuity. Measured in LogMAR (logarithm of the Minimum Angle of Resolution). Change in LogMAR of 0.1 indicates 1 line change on the vision chart

^a^Standardized difference is the mean difference divided by the pooled standard deviation, expressed as a percentage

^b^Cluster effects within school were adjusted for in all comparisons

^c^Defined as having eyeglasses at school at baseline, having previously been told to bring them

^d^Defined as being enrolled in a treatment school that received free eyeglasses

The left column of Table 3 shows the lack of a significant association between self-report of practicing eye exercises regularly and change in LogMAR uncorrected visual acuity at 9 months (− 0.009 [95% CI (− 0.028, 0.010), *p* = 0.336] and 21 months (− 0.011 [95% CI (− 0.031, 0.010), *p* = 0.320], adjusting for baseline VA and other characteristics in the full sample without PSM. Using PSM in the sub-sample of matched children, the results in the right column are similar. No association between eye exercises and change in LogMAR uncorrected VA was found at 9 months (− 0.011 [95% CI (− 0.029, 0.007), *p* = 0.232]) or 21 months (− 0.012 [95% CI (− 0.029, 0.005), *p* = 0.168]).

**Table 3:** Effect of self-reported compliance with eye exercises on change in uncorrected visual acuity between baseline and 9 and 21 months

	Full Sample^a^				Matched Sample^b^			
Outcome	Regularly Practiced Eye Exercises	Did Not Regularly Practice Eye Exercises	Differences Between Means (95% CI)	*P* Value	Regularly Practiced Eye Exercises	Did Not Regularly Practice Eye Exercises	Differences Between Means (95% CI)	*P* Value
9-month uncorrected visual acuity change (LogMAR)	0.11 (0.19)	0.12 (0.18)	−0.009 (− 0.028, 0.010)	0.336	0.11 (0.18)	0.12 (0.19)	− 0.011 (− 0.029, 0.007)	0.232
21-month uncorrected visual acuity change (LogMAR)	0.19 (0.20)	0.21 (0.21)	−0.011 (− 0.031, 0.010)	0.320	0.19 (0.20)	0.21 (0.21)	− 0.012 (− 0.029, 0.005)	0.168

^a^Age, sex, myopia power (diopters of most myopic eye), uncorrected visual acuity (LogMAR of better seeing eye), ownership of eyeglasses, and time spent on near work, middle distance activities and outdoor activity were included in the multiple regression

^b^Matched using propensity score modeling on age, sex, myopia power (diopters of most myopic eye), uncorrected visual acuity (LogMAR of better seeing eye), ownership of eyeglasses, and time spent on near work, middle distance activities and outdoor activity

The left column of Table 4 indicates the lack of a significant association between self-reported participation in eye exercises and eyeglasses wear at 9 months (0.209 [95% CI (− 0.085, 0.503), *p* = 0.163]) and 21 months (− 0.082 [95% CI (− 0.367, 0.203), *p* = 0.572]), using logistic regression adjusted for baseline eyeglasses wear and other baseline characteristics in the full sample without PSM. Results were similar in the PSM analysis: no association between regular eye exercises and the rate of eyeglasses wear was found at 9 months (0.027 [95% CI (− 0.019, 0.072), *p* = 0.252]) or 21 months (− 0.010 [95% CI (− 0.054, 0.034), *p* = 0.666]).

**Table 4:** Effect of eye exercises on rates of eyeglasses wear at 9 and 21 months

	Full Sample^a^				Matched Sample^b^			
Outcome	Regularly Practiced Eye Exercises	Did Not Regularly Practice Eye Exercises	Differences Between Means (95% CI)	*P* Value	Regularly Practiced Eye Exercises	Did Not Regularly Practice Eye Exercises	Differences Between Means (95% CI)	*P* Value
9-month proportion of wearing eyeglasses	0.34 (0.47)	0.32 (0.47)	0.209 (−0.085, 0.503)	0.163	0.35 (0.49)	0.32 (0.47)	0.027 (−0.019, 0.072)	0.252
21-month proportion of wearing eyeglasses	0.69 (0.46)	0.70 (0.46)	−0.082 (− 0.367, 0.203	0.572	0.69 (0.46)	0.70 (0.46)	−0.010 (− 0.054, 0.034)	0.666

^a^Age, sex, myopia power (diopters of most myopic eye), uncorrected visual acuity (LogMAR of better seeing eye), ownership of eyeglasses, and time spent on near work, middle distance activities and outdoor activity were included in the multiple regression

^b^Matched using propensity score modeling on age, sex, myopia power (diopters of most myopic eye), uncorrected visual acuity (LogMAR of better seeing eye), ownership of eyeglasses, and time spent on near work, middle distance activities and outdoor activity

## Discussion

Our finding that the self-reported regular practice of traditional Chinese eye exercises has no protective effect with regard to visual acuity is consistent with the majority of recent reports [11, 23]. A sufficiently-powered randomized controlled trial would be needed to provide a more definitive answer. However, in view of widespread use of eye exercises in schools across China, and existing Chinese government policy supporting the practice, it is unlikely that randomization of children to a control group not performing exercises would be considered ethical, and it is not clear there would be support for such studies outside China. A previous study with small sample size (*n* = 190 children) has reported a statistically significant association between correctly-performed eye exercises and a reduction in accommodative lag in children [10]. Accommodative lag is defined as the difference between accommodative demand and accommodative response, and may be associated with onset and progression of myopia [24]. However, whether a reduction in accommodative lag is an important indicator of long-term myopia risk remains unclear. Given the lack of compelling evidence in the published literature, and practical impediments to trials in China, prospective studies using analytic techniques such as PSM may provide the best-available evidence on the efficacy of Chinese eye exercises in the control of myopia. This issue is of practical importance in the sense that substantial resources expended in China on the promotion of eye exercises might be devoted to other proven interventions to prevent or delay myopia, such as increased outdoor time [25], if in fact the exercises are not effective. Recent increases in myopia prevalence in Chinese children despite the widespread use of eye exercises are consistent with the hypothesis that these exercises, at least as performed, are not fully effective in controlling myopia [26].

Our study failed to find evidence for the hypothesis that performance of eye exercises might be associated with lower rates of eyeglasses wear. This hypothesis was based on the idea that children relying on preventive methods against myopia might be less inclined to comply with myopia treatments such as eyeglasses. In view of the apparent lack of efficacy of exercises in preventing myopia in this setting, it is encouraging that their use did not stand in the way of eyeglasses wear, a proven mode of correcting myopia and improve vision.

Biological plausibility for the effectiveness of Chinese eye exercises on prevention of myopia is low from the standpoint of western medicine. It not clear how periorbital massage would connect with currently-understood pathways controlling myopia progression, such as emmetropization [27], peripheral defocus [28] and retinal dopamine feedback [29].

Strengths of the current study include its large size, inclusion of a population-representative sample drawn from over 250 schools, and the use of a novel statistical approach in this area, PSM. The 21 months follow up period is also among the longest reported in the literature on this issue. Weaknesses must also be acknowledged. Change in uncorrected visual acuity was used as a surrogate for myopia progression. Visual acuity may be affected by multiple subjective and environmental factors, such as ocular fatigue and light. A better indicator for myopia would be the refractive error of children, but in the context of this study accurate refractive error measurements were not available. Vision is also important to the functioning and quality of life of a child. In the Chinese school setting, some 90% of vision impairment is due to uncorrected myopia, but other causes such as amblyopia and refractive errors such as astigmatism likely also contributed to decrements in uncorrected visual acuity, leading to a degree of inaccuracy in our results. To the extent that eye exercises are ineffective against these less common conditions, this may have led to an under-estimation of the effectiveness of exercises against myopia. Further, our assessment of the key independent variable in this study, performance of eye exercises, is based solely on self-report of students, whose interpretation of the term “regular use” may have been subjective and subject to recall bias, leading to imprecision. Lastly, self-reported data cannot ensure that eye exercises were performed properly from the perspective of traditional Chinese medicine. However, the point of the current study is to determine whether eye exercises, as students conduct them in schools, affect visual acuity and eyeglasses wear.

## Conclusion

Despite these weaknesses, and the important limitations inherent as mentioned above in its observational design, this study does add to a growing body of evidence casting doubt on the efficacy of eye exercises, a widely-used modality to prevent myopia in Chinese children. Health workers and administrators should promote eyeglasses wear rather than eye exercises alone to address myopia among rural students.

## Supplementary information

**Additional file 1.** Survey questionnaire in Chinese. Chinse survey questionnaire used in this study.

**Additional file 2.** Survey questionnaire in English. Survey questionnaire used in this study, translated into English.

## Data Availability

The dataset used and/or analyzed during the current study are available from the corresponding author on reasonable request.

## Abbreviations

ETDRS: Early Treatment Diabetic Retinopathy Study
LogMAR: Logarithm of The Minimal Angle of Resolution
PSM: Propensity Score Matching
VA: Visual Acuity
ZOC: Zhongshan Ophthalmic Center of Sun Yat-sen University
